# Supplementary material for: Successful nutritional control of scratching and clinical signs associated with adverse food reaction: A randomized controlled COSCAD'18 adherent clinical trial in dogs in the United States
Source: J Vet Intern Med. 2021 Jun 11;35(4):1884–92. doi: 10.1111/jvim.16193 (PMC8295670; doi:10.1111/jvim.16193)

Table S1: *Macronutrient content of study foods reported on a dry matter basis. PCF= positive control food, TTF= therapeutic test food.*

	PCF	TTF
**Caloric Content (kcal/kg)**	4833	5139
**Crude Protein (%)**	19	17
**Crude Fat (%)**	17	19
**Crude Fiber (%)**	2.5	1.3
**Ash (%)**	8.8	4.8

Table S2: *Ingredient lists of both therapeutic and positive control study foods*.

Study Food	Ingredient List
**Therapeutic Test Food (TTF)**	Brown rice, brewers rice, egg product, rice protein concentrate, soybean oil, flaxseed, chicken flavor, dried beet pulp, fish oil, coconut oil, lactic acid, dicalcium phosphate, potassium chloride, calcium carbonate, lipoic acid, iodized salt, vitamins (vitamin E supplement, L-ascorbyl-2-polyphosphate (source of vitamin C), niacin supplement, thiamine mononitrate, vitamin A supplement, calcium pantothenate, riboflavin supplement, biotin, vitamin B12 supplement, pyridoxine hydrochloride, folic acid, vitamin D3 supplement), green peas, taurine, apples, cranberries, choline chloride, carrots, DL-methionine, natural flavors, minerals (ferrous sulfate, zinc oxide, copper sulfate, manganous oxide, calcium iodate, sodium selenite), mixed tocopherols for freshness, broccoli, beta-carotene.
**Positive Control Food (PCF)**	Corn starch, hydrolyzed poultry by-products aggregate, coconut oil, soybean oil, natural flavors, potassium phosphate, powdered cellulose, calcium carbonate, sodium silico aluminate, chicory, L-tryrosine, fructooligosaccharides, fish oil, L-lysine, choline chloride, taurine, L-tryptophan, vitamins [DL-alpha tocopherol (source of vitamin E), inositol, niacin supplement, L-ascorbyl-2-polyphosphate (source of vitamin C), D-calcium pantothenate, biotin, pyridoxine hydrochloride (vitamin B6), riboflavin supplement, thiamine mononitrate (vitamin B1), vitamin A acetate, folic acid, vitamin B12 supplement, vitamin D3 supplement], DL-methionine, marigold extract (Tagetes erecta L.), histidine, trace minerals (zinc proteinate, zinc oxide, ferrous sulfate, manganese proteinate, copper proteinate, copper sulfate, manganous oxide, calcium iodate, sodium selenite), rosemary extract, preserved with mixed tocopherols and citric acid.

Table S3: *Patient demographics for adult dogs diagnosed with adverse reaction to food. Data are represented as absolute counts or as mean ± SD. †† = one animal was not castrated/spayed. PCF= positive control food, TTF= therapeutic test food*

	**Completed**	**PCF**	**TTF**
**Total**	32	18	14
**Age (yr)**	5.8 ± 3.4	6.6 ± 3.6	5.2 ± 3.2
**Weight (kgs)**	25.2 ± 12	25.7 ± 12	24.6 ± 12
**Gender**			
**Male**	15 (47%)††	8 (44%)	7 (50%)††
**Female**	17 (53%)††	10 (56%)††	7 (50%)
**Breed**			
**Purebred**	20 (63%)	13 (72%)	7 (50%)
**Mixed**	10 (31%)	4 (22%)	6 (43%)
**Not Reported**	2 (6%)	1 (6%)	1 (7%)

Table S4: *Details of concurrent medications that were changed during the study and which may have affected clinical signs.*

Group	***Patient***	***Day 0***	***Day 21***	***Day 42***	***Medication Name***	***Medication Start***	***Medication Finish***
Control	1	9/13/19	10/1/19	10/22/19	Oclacitinib	10/9/19	Ongoing
	2	10/22/19	11/12/19	12/12/19	Terbinafine, mometasone furoate, florfenicol aural solution	10/22/19	10/22/19
	2	10/22/19	11/12/19	12/12/19	Terbinafine, mometasone furoate, florfenicol aural solution	11/25/19	11/25/19
Test	3	12/10/19	1/8/20	1/31/20	Terbinafine, mometasone furoate, florfenicol aural solution	1/8/20	1/8/20
	4	10/3/19	11/6/19	12/4/19	Diphenhydramine	10/13/19	10/16/19

Figure S1: *CONSORT Flow Diagram of patient screening, exclusion, and withdrawals.*

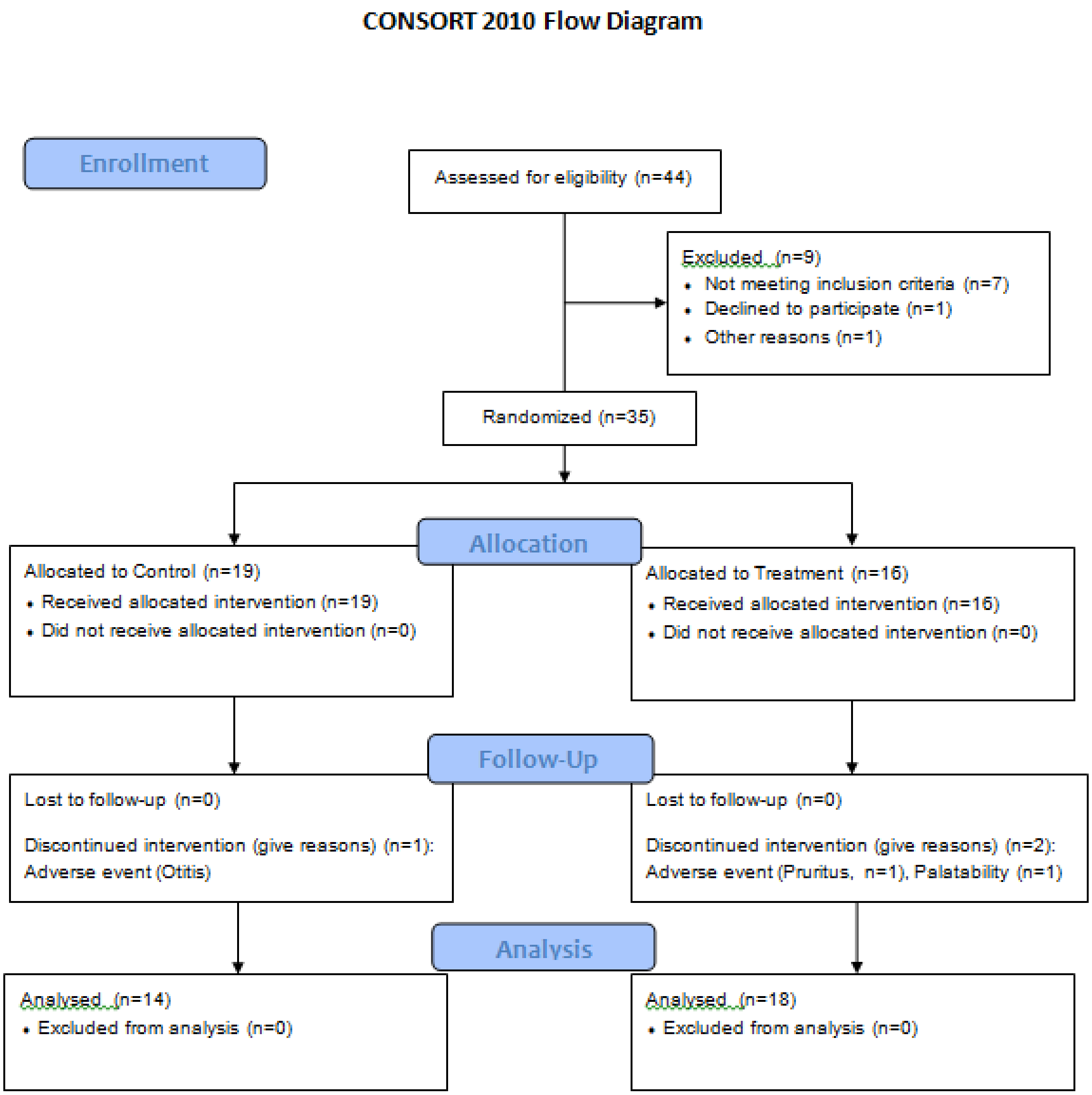

Figure S2: *Comparison of CADLI (A) and PVAS (B) scores between therapeutic test food (TTF) and positive control food (PCF) at Day 0, Day 21 (Baseline), and Day 42 (Treatment). Data are expressed as mean ± SD. CADLI: PCF vs. TTF: mean ± SD: Day 0 = 6 ± 6 vs. 6 ± 7, Day 21 = 6 ± 5 vs. 5 ± 5, Day 42 = 4 ± 4 vs. 4 ± 4; p>.05. PVAS: PCF vs. TTF: mean ± SD: Day 0 = 4.0 ± 2.1 vs. 5.0 ± 2.7, Day 21 = 4.0 ± 2.6 vs. 5.1 ± 2.2, and Day 42 = 3.8 ± 2.7 vs. 4.3±2.2; p>.05).*

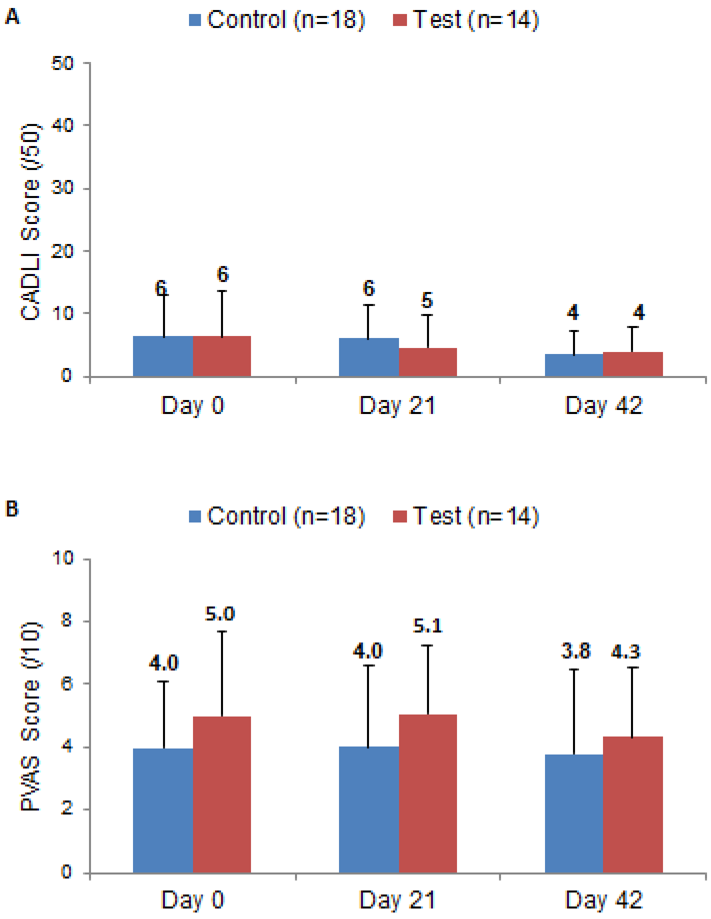

Supplement: Supplementary file 1 — Table S1 Macronutrient content of study foods reported on a dry matter basis. Table S2. Ingredient lists of both therapeutic and positive control study foods. Table S3. Patient demographics for adult dogs diagnosed with adverse reaction to food. Table S4. Details of concurrent medications that were changed during the study and which may have affected clinical sign. Figure S1. CONSORT flow diagram of patient screening, exclusion, and withdrawals. Figure S2. Comparison of CADLI (A) and PVAS (B) scores between therapeutic test food (TTF) and positive control food (PCF) at Day 0, Day 21 (baseline), and Day 42 (treatment). [file JVIM-35-1884-s001.pdf]
